# Cloning, characterization, and expression of microRNAs from the Asian malaria mosquito, *Anopheles stephensi*

**DOI:** 10.1186/1471-2164-9-244

**Published:** 2008-05-23

**Authors:** Edward Andrew Mead, Zhijian Tu

**Affiliations:** 1 Department of Biochemistry, Virginia Polytechnic Institute and State University, Blacksburg, VA 24061, USA

## Abstract

**Background:**

microRNAs (miRNAs) are non-coding RNAs that are now recognized as a major class of gene-regulating molecules widely distributed in metozoans and plants. miRNAs have been found to play important roles in apoptosis, cancer, development, differentiation, inflammation, longevity, and viral infection. There are a few reports describing miRNAs in the African malaria mosquito, *Anopheles gambiae*, on the basis of similarity to known miRNAs from other species. *An. stephensi *is the most important malaria vector in Asia and it is becoming a model *Anopheline *species for physiological and genetics studies.

**Results:**

We report the cloning and characterization of 27 distinct miRNAs from 17-day old *An. stephensi *female mosquitoes. Seventeen of the 27 miRNAs matched previously predicted *An. gambiae *miRNAs, offering the first experimental verification of miRNAs from mosquito species. Ten of the 27 are miRNAs previously unknown to mosquitoes, four of which did not match any known miRNAs in any organism. Twenty-five of the 27 *Anopheles *miRNAs had conserved sequences in the genome of a divergent relative, the yellow fever mosquito *Aedes aegypti*. Two clusters of miRNAs were found within introns of orthologous genes in *An. gambiae, Ae. aegypti*, and *Drosophila melanogaster*. Mature miRNAs were detected in *An. stephensi *for all of the nine selected miRNAs, including the four novel miRNAs (miR-x1- miR-x4), either by northern blot or by Ribonuclease Protection Assay. Expression profile analysis of eight of these miRNAs revealed distinct expression patterns from early embryo to adult stages in *An. stephensi*. In both *An. stephensi *and *Ae. aegypti*, the expression of miR-x2 was restricted to adult females and predominantly in the ovaries. A significant reduction of miR-x2 level was observed 72 hrs after a blood meal. Thus miR-x2 is likely involved in female reproduction and its function may be conserved among divergent mosquitoes. A mosquito homolog of miR-14, a regulator of longevity and apoptosis in *D. melanogaster*, represented 25% of all sequenced miRNA clones from 17-day old *An. stephensi *female mosquitoes. *An. stephensi *miR-14 displayed a relatively strong signal from late embryonic to adult stages. miR-14 expression is consistent during the adult lifespan regardless of age, sex, and blood feeding status. Thus miR-14 is likely important across all mosquito life stages.

**Conclusion:**

This study provides experimental evidence for 23 conserved and four new microRNAs in *An. stephensi *mosquitoes. Comparisons between miRNA gene clusters in *Anopheles *and *Aedes *mosquitoes, and in *D. melanogaster *suggest the loss or significant change of two miRNA genes in *Ae. aegypti*. Expression profile analysis of eight miRNAs, including the four new miRNAs, revealed distinct patterns from early embryo to adult stages in *An. stephensi*. Further analysis showed that miR-x2 is likely involved in female reproduction and its function may be conserved among divergent mosquitoes. Consistent expression of miR-14 suggests that it is likely important across all mosquito life stages from embryos to aged adults. Understanding the functions of mosquito miRNAs will undoubtedly contribute to a better understanding of mosquito biology including longevity, reproduction, and mosquito-pathogen interactions, which are important to disease transmission.

## Background

microRNAs (miRNAs) are non-coding RNAs that are now recognized as a major class of gene-regulating molecules widely distributed in metozoans and plants [1,2]. Many miRNA genes are transcribed by RNA polymerase II, yielding primary miRNAs (pri-miRNAs) of hundreds to thousands of bases in length [1,3-5]. A given pri-miRNA can be either monocistronic, containing a sequence for one mature miRNA, or polycistronic, containing a sequence for multiple mature miRNA products [1,3,6]. In *Drosophila*, the pri-miRNA is processed by a Drosha-Pasha complex to yield pre-miRNA, small stem-loop structures that are approximately 70 nucleotides (nt) in length [7,8]. These stem-loops, or hairpins, are then exported to the cytoplasm and processed by Dicer-1 to form an miRNA:miRNA* duplex [1,3,6]. The duplex molecules are separated by a helicase, and based upon the strength of 5' end pairing, one single strand is chosen as the mature miRNA [3]. The opposing strand is referred to as the miRNA* strand, and believed to rapidly degrade following separation [3]. Mature miRNAs associate with an Argonaute protein and bind their mRNA targets, which are often in the 3' untranslated region (UTR), resulting in inhibition of translation or possibly target mRNA degradation in animals [2]. The "seed region" (bases 2–8 from the 5' end) contributes significantly to miRNA-target recognition [9,10].

miRNAs have been found to play important roles in apoptosis, cancer, development, differentiation, inflammation, longevity, and viral infection [8,11-17]. Estimates of the extent of miRNA gene regulation vary from 4% of transcripts in the *Drosophila *ovary [18] to a third of human genes [19]. It is estimated that approximately 110 different miRNAs are expressed across the different life stages of *D. melanogaster *[20]. In flies, the adult stage is characterized by significant miRNA expression [21]. In a study to uncover *Drosophila *miRNAs, Lai *et al*. (2003) reported 38 putative miRNAs in the African malaria mosquito, *Anopheles gambiae*, that are conserved with *Drosophila *miRNAs [20]. There are two additional reports describing *An. gambiae *miRNAs on the basis of similarity to known miRNAs [22,23]. However, there is no direct experimental evidence for any of these miRNAs in mosquitoes. We are interested in identifying conserved as well as mosquito-specific microRNAs and exploring their potential functions in mosquito biology and mosquito-pathogen interactions. We carried out our cloning work on 17-day old adult female mosquitoes, which are highly relevant to disease transmission because it takes approximately two weeks for *Plasmodium *parasites to mature and become infective within a female mosquito [24]. We used *An. stephensi *because this species is an important malaria vector in Asia and it is becoming a model *Anopheline *species for physiological and genetics studies. Here we report direct cloning and characterization of 23 conserved and four new miRNAs from the *An. stephensi *adult female. Comparative analysis uncovered the loss or significant change of two miRNA genes in a divergent mosquito *Ae. aegypti*. We also determined the expression profile of several selected miRNAs including the four new miRNAs across all life stages of *An. stephensi*. We performed further expression analysis on two miRNAs that are implicated in mosquito reproduction and longevity.

## Results

One hundred and forty-eight *An. stephensi *small RNA sequences showed 100% match to the *An. gambiae *genome assembly and were identified as probable miRNA sequences (Table 1). These small RNAs were represented by 27 distinct sequences (Table 2). Thirteen additional small RNA sequences had 1 mismatch to the *An. gambiae *genome assembly (see Additional file 1). To be conservative, we only considered these 13 sequences as possible miRNA candidates (Table 1 and Additional file 1) and we did not include them in our list of *Anopheles *miRNAs.

**Table 1:** Classification of Cloned Small RNAs in *An. stephensi*.

**RNA Species**	**Number Present**	**% of Total Clones**
miRNAs shown in Table 2 ^1^	148	40.22%
Possible miRNAs listed in Additional file 1^1^	13	3.53%
rRNA	3	0.82%
tRNA	16	4.35%
Unidentified ^2^	51	13.86%
Low Quality or Short Sequences ^3^	137	37.23%
**TOTAL**	**368**	**100%**

1. We decided to include only miRNA candidates that match 100% to a locus in the *An. gambiae *genome as true miRNA candidates shown in Table 2. There are several additional miRNA candidates that have 1 nucleotide mismatch to loci in the *An. gambiae *genome, which could either result from real differences between *An. stephensi *and *An. gambiae*, or errors introduced during cloning or sequencing. These miRNA candidates are not included in Table 2 but are provided in Additional file 1 as we feel further investigation is necessary to ascertain their identities.

2. Sequences do not match any known miRNAs or any other small RNAs or mRNAs.

3. Low quality sequences and sequences less than 17 nucleotides were not analyzed further.

**Table 2:** Sequence and Characteristics of Cloned miRNAs in *An. stephensi*.

**miRNA **^1^	**miRBase Name**^3^	**Sequence**^4^	**Occurrence **^5^	**Location**^6^	**Score **^7^
ast-let-7 (Ia)	ast-let-7	UGAGGUAGUUGGUUGUAUAGU	12	3R, 10270763 (-)	14.8
ast-miR-124 (Ia)	ast-miR-124	UAAGGCACGCGGUGAAUGC	1	3R, 29002032 (+)	11.32
ast-miR-14 (Ia)	ast-miR-14	UCAGUCUUUUUCUCUCUCCUA	38	3R, 24898098 (+)	16.9
ast-miR-210 (Ia)	ast-miR-210	UUGUGCGUGUGACAACGGCUA	7	X, 21450383 (+)	12.72
ast-miR-276 (Ia)	ast-miR-276-5p	AGCGAGGUAUAGAGUUCCUA	4	2L, 18991766 (+)	15.96
ast-miR-277 (Ia)	ast-miR-277	UAAAUGCACUAUCUGGUACGA	4	2R, 28234532 (-)	15.78
ast-miR-277* ^2^ (Ia)		CGUGUCAGAGGUGCAUUUA	1	2R, 28234583 (-)	15.78
ast-miR-281 (Ia)	ast-miR-281	UGUCAUGGAAUUGCUCUCUUUA	24	2L, 17362444 (-)	16.14
ast-miR-283 (Ia)	ast-miR-283	AAAUAUCAGCUGGUAAUUCU	1	2R, 37890092 (-)	14.92
ast-miR-317 (Ia)	ast-miR-317	UGAACACAUCUGGUGGUAUCU	10	2R, 28252007 (-)	10.57
ast-miR-8 (Ia)	ast-miR-8	UAAUACUGUCAGGUAAAGAUGU	13	3L, 38943098 (+)	14.73
ast-miR-8* ^2^ (Ia)		CAUCUUACCGGGCAGCAUUA	1	3L, 38943058 (+)	14.73
ast-miR-9a (Ia)	ast-miR-9a	UCUUUGGUUAUCUAGCUGUAU	3	2L, 15089338 (-)	13.6

ast-miR-11 (Ib)	ast-miR-11	CAUCACAGUCUGAGUUCUUGCU	1	2R, 13042084 (-)	14.97
ast-miR-276a (Ib)	ast-miR-276-3p	UAGGAACUUCAUACCGUGCUCU	2	2L, 18991809 (+)	14.72
ast-miR-34 (Ib)	ast-miR-34	UGGCAGUGUGGUUAGCUGGUU	5	2R, 28232720 (-)	17.77 ^7^
ast-miR-87 (Ib)	ast-miR-87	GGUGAGCAAAUAUUCAGGUGU	1	X, 261196 (-)	12.12

ast-miR-12 (IIa)	ast-miR-12	UGAGUAUUACAUCAGGUACUGGU	2	2R, 37888125 (-)	8.45
ast-miR-375 (IIa)	ast-miR-375	UUUGUUCGUUUGGCUCGAGUUA	1	3R, 51640581 (-)	10.44
ast-miR-2a (IIa)	ast-miR-2-1	UAUCACAGCCAGCUUUGAUGAG	2	2L, 37757111 (-)	15.99

ast-miR-304 (IIb)	ast-miR-1889	ACACAUUACAGAUUGGGAUUA	2	2R, 37888805 (-)	NS ^8^
ast-miR-306 (IIb)	ast-miR-306	UCAGGUACUGGAUGACUCU	1	3R, 5888649 (-)	7.53 ^8^
ast-miR-76 (IIb)	ast-miR-981	UUCGUUGUCGACGAAACCUG	2	X, 1228349 (+)	12.73

ast-miR-x1 (IIc)	ast-miR-996	UGACUAGAUUACAUGCUCGU	1	2R, 55572846 (-)	16.19
ast-miR-x2 (IIc)	ast-miR-989	AUGUGAUGUGACGUAGUGGUA	6	3L, 2905484 (+)	15.15
ast-miR-x3 (IIc)	ast-miR-1890	UGAAAUCUUUGAUUAGGUCU	1	3R, 21181098 (-)	17.45
ast-miR-x4 (IIc)	ast-miR-1891	UGAGGAGUUAAUUUGCGUGUUUU	2	3R, 5819094 (-)	14.40

1. The names for these *An. stephensi *miRNAs are temporarily assigned according to similarity to known miRNAs. Formal name assignment will be made after miRBase submission. ast stands for *An. stephensi*. *An. stephensi *miRNAs shown here are divided into two main categories. Category I includes miRNAs that match *An. gambiae *miRNA predictions that were previously reported in miRBase (category Ia) or [23], category Ib). Category II are miRNAs that have not been reported in *An. gambiae *or any other mosquito species. This category includes miRNAs that displayed perfect or near-perfect (1 nucleotide mismatch only) match to miRNAs from non-mosquito species in miRBase (category IIa), miRNAs that displayed 80% or higher overall similarity to miRNAs from non-mosquito species in the miRBase (category IIb), as well as miRNAs that showed no match to any miRNAs in miRBase at the default e-value cutoff of 10 (category IIc). miRNAs in category IIc are temporarily labeled with an "x" in front of an Arabic numeral.

2. A "*" delineates that the sequence matches the miRNA* strand of miRNA:miRNA* heteroduplex.

3. These are formal names assigned by miRBase, which were received during the proofing stage. See the last paragraph of the Discussion section for details.

4. The longest sequence of each miRNA is shown. Variants with different ends are shown in Additional file 1. The observation of end variants has literary precedence (see [32]).

5. Occurrence refers to the number of times a sequence appeared during our cloning and sequencing.

6. Location refers to the location of match in the *An. gambiae *genome as there is no *An. stephensi *genome assembly available. The location is indicated by chromosome name, start position of the mature miRNA, and strand orientation. All matches are 100%.

7. Score refers to the result of miRscan analysis. Unless otherwise noted, the pre-miRNA sequences used for miRscan are pairs from *An. gambiae *and *Ae. aegypti*. In the original miRscan paper [29], most of the validated *C. elegans *miRNAs received scores of 9 or above although a small number of them received scores significantly less and some even received negative scores.

8. The precursor sequence of *Ae. aegypti *miR-34 contains a large segment in the loop region, which may be the cause for no score or "NS" by miRscan for the *An. gambiae *and *Ae. aegypti *pre-miR-34 alignment. Thus *An. gambiae *and *D. melanogaster *pre-miR-34 alignment was used for miRscan, which produced a score of 17.99.

9. We could not find homologs for miR-304 and miR-306 in *Ae. aegypti*. Homologs were found in *D. melanogaster *(see Figure 3), which were used for miRscan analysis shown here.

### Confirmation of previously predicted *Anopheline *miRNAs by direct cloning and northern blot

**Figure 1:**
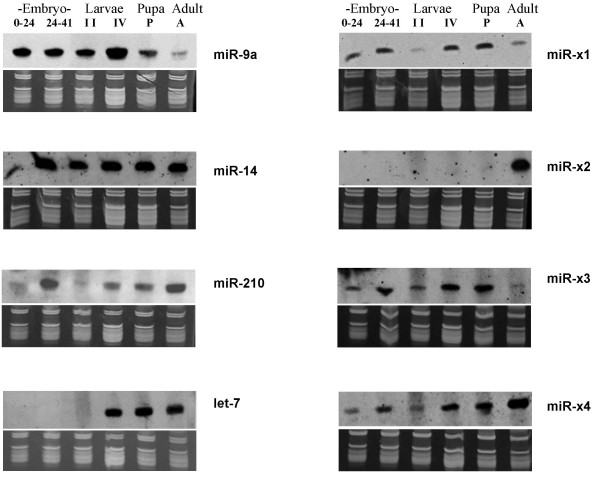
**Northern analysis of eight miRNAs across different developmental stages in *An. stephensi***. Shown here are eight northern blots performed using Dig-labeled miRCURY LNA probes designed for hybridization to either miR-14, let-7, miR-9a, miR-210, or to one of the four novel miRNAs (miR-x1–x4). The top panels are northern results and the bottom panels are RNA gels for verification of small ribosomal and tRNA integrity and equal loading of total RNA. ssDNA size markers (19 and 23 nts, not shown) were also visualized on the RNA gel for size estimation. Ten micrograms of total RNA for each sample were used. Developmental stages examined were early embryo (Embryo 0–24: 0–24 hrs after egg deposition), late embryo (Embryo 24–41: 24–41 hrs after egg deposition), intermediate and late larval stages (II and IV, respectively), Pupa (P), and Adult (A). To be consistent with our cloning experiment, 17-day old adult females were used in these northern experiments.

Seventeen of the 27 *An. stephensi *sequences shown in Table 2 match predicted *An. gambiae *miRNA hairpins (Table 2, category I). Fifteen of the 17 matches coincide with the predicted mature miRNAs described either at miRBase [25] (Table 2, category Ia) or in Chatterjee and Chaudhuri (2006) [23] (Table 2, category Ib). Two sequences appear to be miRNAs*, the passenger strand of the miRNA:miRNA* duplex. The copy numbers of ast-miR-8*, and ast-mir-277* are less than those of ast-miR-8 and ast-miR-277, respectively. Intriguingly, miR-14, which is involved in the regulation of apoptosis and longevity in *D. melanogaster *[26], represents 25% of all the identified miRNAs. Northern analysis using total RNA from 17-day old females with antisense Locked Nucleic Acid (LNA) probes against 4 selected miRNAs (miR-9a, -14, -210, and let-7) all showed bands of the correct size, confirming cloning results (Figure 1, the last lane). The LNA oligos contain a mixture of DNA nucleotides and LNA nucleotides with 2'-4' methylene linkage providing high binding affinity and enhanced specificity to targets as compared to ordinary DNA oligos [27].

### Six new miRNAs in *Anopheles *that are similar to known miRNAs from other organisms

**Figure 2:**
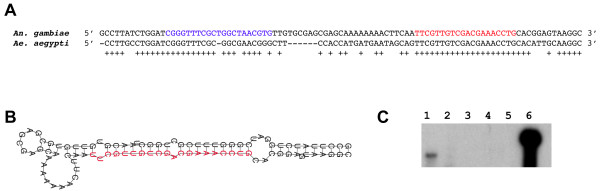
**Analysis of ast-mir-76, a miRNA that was previously unknown in mosquitoes**. The mature miRNA sequence was cloned from *An. stephensi*, and the hairpin precursor sequence was obtained from the *An. gambiae *genome assembly. **A) **Alignment of the pre-miRNA hairpins found in *An. gambiae *and *Ae. aegypti*. The mature miRNA is marked in red while miRNA* is marked in blue. Conserved nucleotides are indicated by a "+". **B) **Hairpin structure of *An. gambiae *mir-76. The mature miRNA predicted by miRscan (Table 2) is shown in red. **C) **RPA analysis of ast-mir-76. Lane 1, *An. stephensi *RNA with probe and digested; Lane 2, yeast RNA with probe and digested; Lane 3, yeast RNA without probe and digested; Lanes 4 and 5, empty lanes; Lane 6, undigested probe. A band of the correct size was only observed in *An. stephensi *total RNA samples (Lane 1). The size of the protected RNA product in lane 1 was estimated to be 24 nucleotides using markers as described in Figure 1. This size is as expected (the protected 20-nt long ast-miR-76 plus 4 undigested adenosines, see Methods).

Three *An. stephensi *small RNAs display perfect or nearly perfect (only 1 mismatch) match to published miRNAs from organisms other than mosquitoes (Table 2, category IIa) and three more show high similarity (> 84% identity over 19 or more nt) to miRNAs from other organisms (Table 2, category IIb). These small RNAs are named according to their closest miRBase matches, which are listed in Additional file 1. All precursor sequences for each of the six miRNAs obtained from the *An. gambiae *genome assembly formed good hairpins (see Figure 2B for an example). Four of the six *Anopheline *miRNAs (miR-12, miR-375, miR-2a, and miR-76) have conserved sequences in *Ae. aegypti *and comparisons between the *Anopheles *and *Aedes *hairpins produced miRscan scores ranging from 8.46 to 15.99, supporting their miRNA status (see Table 2 for the range of expected miRscan scores). miRscan looks for hallmarks of miRNAs within a pair of conserved precursor stem-loop sequences by calculating a score based on seven criteria, the most important of which is the conservation of the base pairing between a miRNA and its antisense [28-30]. Two of the miRNAs, ast-miR-304 and ast-miR-306, do not have conserved sequences (either mature miRNA or hairpin) in *Ae. aegypti *and are described in the next section. We selected ast-miR-76, a miRNA that showed the lowest similarity to known miRNAs in category IIa and IIb, for further verification using Ribonuclease Protection Assay (Figure 2), which is theoretically more sensitive than northern blot (Figure 1). A product of expected size was detected thus supporting the expression of ast-miR-76.

Two of the above miRNAs are worth noting. ast-miR-2a matches dme-miR-2a perfectly thus is named as miR-2a. There is an aga-miR-2 in miRBase (derived from two precursors aga-miR-2-1 and aga-miR-2-2), which is reverse complementary to ast-miR-2a. Sequence comparison showed that ast-miR-2a is not derived from aga-miR-2* because of the existence of multiple indels/mismatches between the alignment of aga-miR-2 and aga-miR-2*. The orientation of our cloned ast-mir-2a is consistent with the miRscan prediction based on *An. gambiae *and *Ae. aegypti *hairpins (Table 2, score 15.99). In addition, ast-mir-304 is reverse complementary to its top match (dme-miR-304) with 86% identity, which is described in the next section.

### *Anopheles *miR-304 and miR-306 are in two separate miRNA clusters located in introns of protein-coding genes

**Figure 3:**
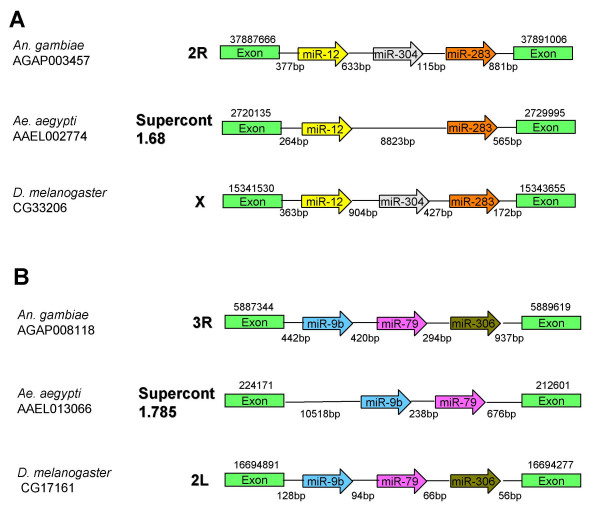
**Clustering of miRNAs genes**. **A) **A miRNA gene cluster within an intron of a conserved gene of unknown function. The miRNA gene cluster contains miR-12, -283, and -304. **B) **A miRNA gene cluster within an intron of a gene coding for a serine-threonine kinase group protein. The miRNA gene cluster contains miR-9b, -79, and -306. Note that one miRNA was not found in the genome of *Ae. aegypti *in both panels. Species name and gene identification are provided at the left side of the figure. Chromosome or supercontig numbers are indicated right next to diagram depicting the miRNA gene clusters. Chromosomal or supercontig positions of the regions depicted are above the boxes showing the exons. miRNA genes are shown as open arrows. The distance between the miRNA genes and neighboring exons are indicated below the diagram. The figure is not drawn to scale. The exons shown in both panels are orthologous as indicated by conserved amino acid sequences.

As mentioned above, *Anopheles *miR-304 and miR-306 do not have conserved sequences in *Ae. aegypti*. However, they both have homologs in *D. melanogaster *(Figure 3). Comparisons between miR-306 sequences in *An. gambiae *and *D. melanogaster *produced a miRscan score of 7.53, which is consistent with its miRNA status. Comparisons between miR-304 sequences in *An. gambiae *and *D. melanogaster *produced no score (NS) during miRscan analysis. The failure to produce a positive score by miRscan does not automatically indicate that miR-304 is not a true miRNA because nine out of the 88 known *C. elegans/C. briggsae *miRNAs produced no scores and two even gave negative scores [29]. A closer examination of the miR-304 hairpin from *An. gambiae *suggested that it met all of the previously described criteria for miRNA structures [31,32].

Interestingly, miR-304 is closely flanked by miR-12 and miR-283 while miR-306 is in a different cluster with miR-9b and miR-79 (Figure 3). Clustering of *An. gambiae *miR-9b and miR-79 is noted on miRBase, but not miR-306; clustering of *An. gambiae *miR-304, -12, and -283 is not predicted in miRBase [33,34]. Both clusters are within introns of protein coding genes. The miR-12, -304, -283 cluster occurs within a conserved gene of unknown function, while the miR-9b, -79, -306 cluster occurs within an ortholog of a gene coding for a *Drosophila *serine-threonine kinase group protein. The exons flanking each of the miRNA clusters are conserved between *An. gambiae, Ae. aegypti*, and *D. melanogaster*, which indicates that the clusters are orthologous. The order of miRNAs within the introns is conserved between *An. gambiae *and *D. melanogaster*, again supporting the miRNA status of the *Anopheles *miR-304 and miR-306. All miRNAs in the two clusters are in the same orientation as the flanking genes, indicating that these miRNAs may be transcribed from the promoters of their respective flanking genes, which is consistent with previous reports [35,36]. In this regard, it is suggestive that, albeit reverse complementary to each other, the mature miR-304 in *Anopheles *and *Drosophila *are both correctly annotated because the orientation of transcription of miR-304 is consistent with the flanking gene in both species.

### Discovery of four novel mosquito miRNAs that have no apparent homolog outside of mosquitoes

**Figure 4:**
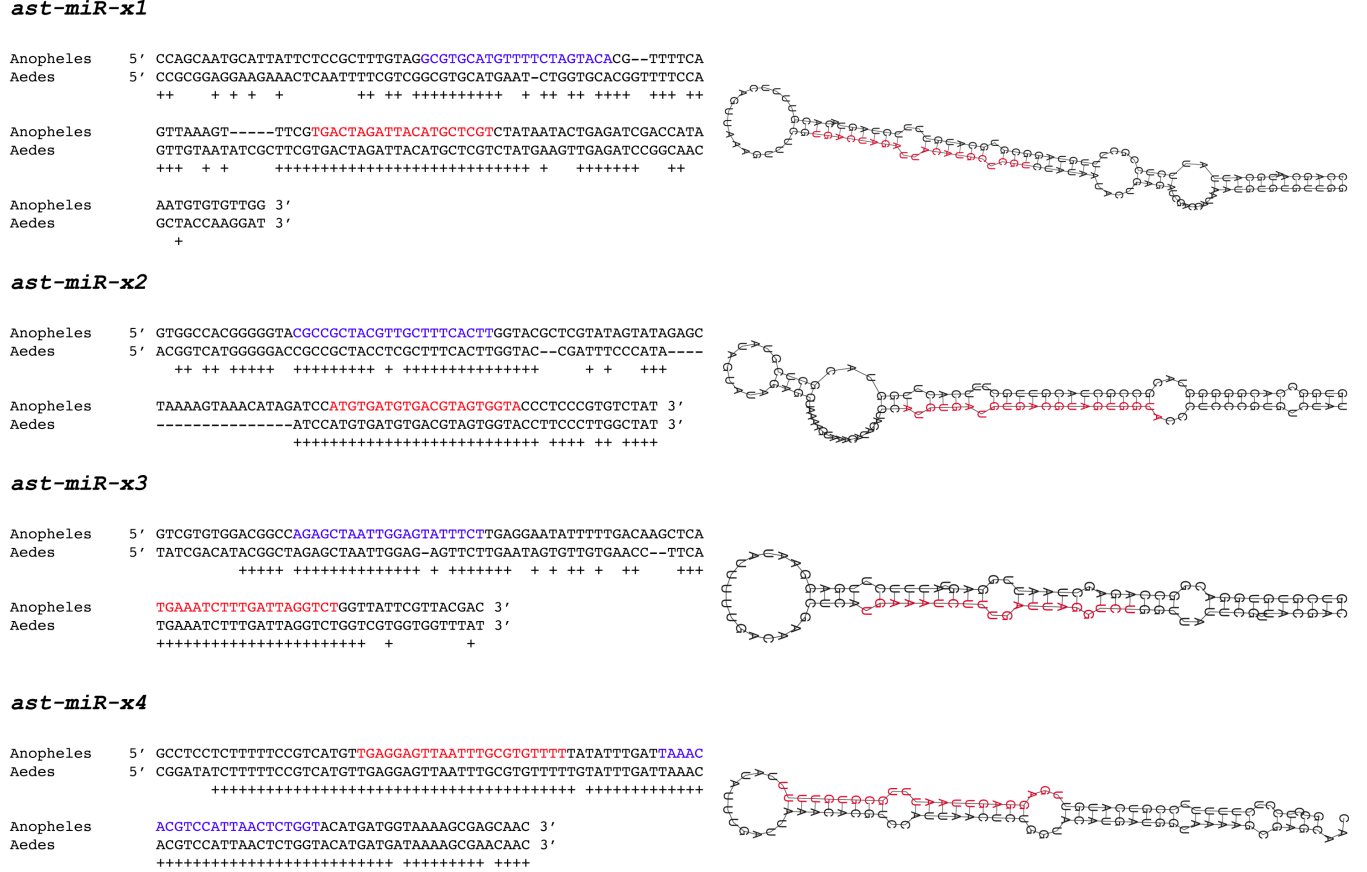
**Sequence alignment and predicted secondary structure of four novel miRNAs**. Shown on the left are the sequence alignments between *An. gambiae *and *Ae. aegypti *miRNA precursor hairpins. Plus signs indicate conservation. The mature miRNA is marked in red while miRNA* is marked in blue. Note the perfect conservation of the mature miRNA (red), high conservation of the miRNA* sequence (blue), and lower conservation of the surrounding stem and loop structure, a hallmark conservation pattern of pre-miRNAs. Shown on the right are the predicted secondary structures of corresponding *An. gambiae *miRNA hairpins. The mature miRNA is marked in red on the hairpin.

As shown in category IIc of Table 2, there are four *An. stephensi *small RNAs that produced no hits to any known miRNAs from any species based upon searches of the miRBase with an e-value cutoff of 10. These sequences are temporarily named ast-miR-x1 through ast-miR-x4. All four sequences match perfectly to unique locations in the *An. gambiae *genome assembly. Putative precursor sequences flanking the four miRNAs in *An. gambiae *showed strong hairpin structures (Figure 4). The precursors of all four miRNAs showed high similarity to *Ae. aegypti *genomic sequences and these hairpin pairs gave miRscan scores between 14.4–17.45, indicating that they strongly resemble the structure and conservation pattern of known miRNAs. In particular, the mature miRNA sequences were 100% conserved between *An. gambiae, An. stephensi*, and *Ae. aegypti*. Northern blot analysis using 17-day old female samples provided further confirmation for these four miRNAs (Figure 1, last lane).

### Expression profile of mosquito miRNAs across different developmental stages

**Figure 5:**
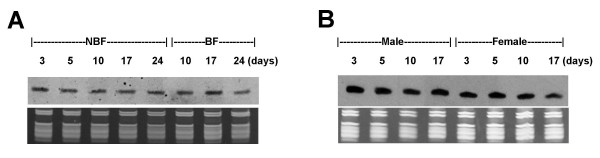
**miR-14 expression across *An. stephensi *adult lifespan**. Shown here are northern blots performed using Dig-labeled miRCURY LNA probes designed for hybridization to miR-14. The top panel is the northern result and the bottom panel is a corresponding RNA gel for verification of small ribosomal and tRNA integrity and equal loading of total RNA. ssDNA size markers (19 and 23 nts, not shown) were also visualized on the RNA gel for size estimation. Ten micrograms of total RNA for each sample were used. **A) **miR-14 expression in *An. stephensi *adult females fed with either sugar water (NBF, non-bloodfed) or blood meal (BF, bloodfed). The samples were 3, 5, 10, 17, and 24 day old adult females that were maintained on sugar water as well as adult females that were fed on blood on day 5 after emergence and collected at day 10, 17, and 24. Bloodfed females were allowed to oviposit two days after the blood meal. **B) **miR-14 expression in *An. stephensi *males and NBF females between 3–17 days of age. We did not extend the comparative analysis to 24 days post emergence because the majority of males do not survive that long.

**Figure 6:**
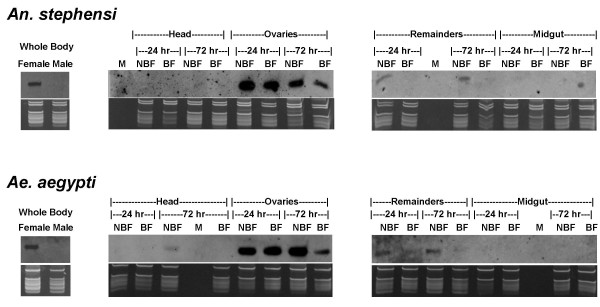
**Expression of miR-x2 in *An. stephensi *and *Ae. aegypti*: sex-specificity, tissue distribution and the impact of blood feeding**. Shown here are northern blots performed using Dig-labeled miRCURY LNA probes designed for hybridization to miR-x2. The top panels are northern results and the bottom panels are RNA gels for verification of small ribosomal and tRNA integrity and equal loading of total RNA. ssDNA size markers (19 and 23 nts, not shown) were also visualized on the RNA gel for size estimation. On the left panel for each species, a comparison between 5-day old adult male and 5-day old non-bloodfed female is shown. Ten micrograms of total RNA isolated from the whole mosquitoes were used. The middle and right panels are comparisons between adult female tissues or body parts in each species. Tissues used were Heads, Ovaries, Midguts, and Remainders. There were four samples for each tissue: BF, tissue sample from bloodfed females at 24 and 72 hrs post-bloodfeeding; NBF, tissue sample from non-bloodfed (sugar-fed) females at equivalent time points compared to the blood-fed samples. Five micrograms of total RNA for each sample were used. The markers lane is designated with an 'M' although the markers are not within the gel image panel because they are below the size of the ribosomal and tRNA.

We decided to expand the expression analysis beyond the adult stage and investigate the expression profiles of eight miRNAs across different developmental stages, from early embryo to female adult. Shown in Figure 1 are northern blot results for four known miRNAs as well as all four new miRNAs. Each miRNA showed a distinct pattern. miR-9a is expressed in all life stages examined and its expression is reduced in adults, which is consistent with what was observed in *D. melanogaster *[21]. The level of miR-210 appears to be higher in late embryo and adult females than in other stages. Let-7 expression begins in late larvae in mosquitoes and continues into adult, again similar to what was observed in *D. melanogaster*. We also determined the expression profile of miR-14, which represents 25% of the miRNA sequences during our cloning experiment (Table 2). As shown in Figure 1, miR-14 displays a strong signal starting from late embryonic to adult stages. miR-14 is observed in all life stages in *D. melanogaster *as well [37]. Further examination of miR-14 expression across adult lifespan showed a relatively consistent expression regardless of age, sex, and hematophagy (Figure 5). The four novel miRNAs (miR-x1, -2, -3, -4) have unique expression patterns as well, which will likely provide useful clues to their function for future research. For example, miR-x2 showed adult-specific expression while miR-x3 showed predominantly pre-adult expression (Figure 1). miR-x2 was not detected in adult males (Figure 6). miR-x3 was at best weakly expressed in adult males while miR-x1 and x4 were clearly expressed in adult males (data not shown).

### miR-x2 in both *An. stephensi *and *Ae. aegypti *is predominantly found in the ovaries and its level is significantly reduced 72 hrs after blood feeding

We decided to carry out a detailed expression analysis of miR-x2 in both *An. stephensi *and *Ae. aegypti*. When different *An. stephensi *tissues were analyzed (Figure 6), miR-x2 showed strong signals in ovaries while no expression was detected in the midgut samples. The expression of miR-x2 was weak in the heads and the "remainders" (thorax plus abdomen without midgut and ovary). Following blood feeding, miR-x2 expression in the ovaries remained high at 24 hours post bloodmeal, but declined sharply by 72 hours post bloodmeal. miR-x2 was hardly detectable among the other tissues in either of the post bloodmeal time points. The same pattern of expression was observed in the distantly related *Ae. aegypti *(Figure 6). We also confirmed the lack of miR-x2 expression in adult males in both *An. stephensi *and *Ae. aegypti *(Figure 6). Thus miR-x2 expression profile is conserved between the two divergent mosquitoes in all samples tested.

## Discussion

Two criteria are critical for demonstrating a valid miRNA [31,38]. First, expression of an approximately 22 nt RNA should be detected by small RNA cloning or by RNA hybridization methods such as northern blot [31,38]. Second, the miRNA should be traceable back to a precursor with a hairpin structure [31,38]. All 27 miRNAs described in this report, including two miRNA* sequences, met the above criteria. The expression was indicated by direct cloning of small RNAs from 17-day old female *An. stephensi *samples and the precursor hairpin was identified using the genome assembly of a related *Anopheles *species of the same subgenus, *An. gambiae*. Additional evidence to validate miRNA status that was proposed in the literature includes proof of processing to a mature form via Dicer, and conservation of the mature sequence and its precursor [31,38]. For all 27 *Anopheles *miRNAs, we were able to identify conserved sequences encompassing the entire hairpin structure either from the genome assembly of a divergent mosquito species, *Ae. aegypti *(25 out of 27) or from *D. melanogaster *(see Additional file 1). Furthermore, miRscan analysis based on conserved hairpin alignment provided strong support for 26 of the 27 Anopheline miRNAs. In the case of ast-miR-304, which has no conserved sequence in *Ae. aegypti *and no miRscan score, a closer examination of the miR-304 hairpin from *An. gambiae *suggests that it meets all of previously described criteria for miRNA structures [31,32]. Furthermore, the *Anopheles *miR-304 and the *D. melanogaster *miR-304 are both in a conserved miRNA cluster and both are flanked by miR-12 and miR-283, thus lending additional support for the validity of *Anopheles *miR-304. Finally miRNA expression was detected for all nine selected ast-miRNAs during either northern blot or RPA, complementing our cloning results. Thus we feel that the overall support for the presence of these 27 miRNAs in *Anopheles *mosquitoes is strong. One miRNA, ast-miR-2a, is worth noting. Cloning results for ast-miR-2a, comparisons to dme-miR-2a, and miRscan predictions are all consistent. However, ast-miR-2a is reverse complementary to the aga-miR-2 reported in miRBase.

As mentioned earlier, miR-304 and miR-306 do not have obvious homologs in *Ae. aegypti*. In both *An. gambiae *and *D. melanogaster*, miR-304 is closely flanked by miR-12 and miR-283 while miR-306 is in a different cluster with miR-9b and miR-79 (Figure 3). Both clusters are in the intron of orthologous genes in the two species. Comparisons of the two miRNAs clusters in these three species (Figure 3) would suggest that miR-304 and miR-306 may have either been lost in *Ae. aegypti *or evolved to significantly different sequences. As the mature miRNA sequences of the *Anopheles *and *Drosophila *miR-304 are reverse complementary to each other, it is possible that the miR-304 precursor sequence may have undergone an inversion after the two dipteran lineages separated. Thus comparisons of clear orthologous miRNA clusters will likely shed new light on the evolution (expansion, loss, rearrangement) of miRNAs, which has not been well studied.

The expression profiles of the eight mosquito miRNAs are informative. When *D. melanogaster *data are available for comparison as in the cases of let-7 and miR-9a, similar expression profiles were found between *An. stephensi *and *D. melanogaster*. This is not surprising as these conserved miRNAs are likely to have similar functions in these Dipteran insects. miR-14 was expressed in the same stages in *An. stephensi *as in *D. melanogaster*. We provided extended expression analysis on miR-14, the miRNA that represents 25% of the sequenced miRNAs during the cloning of the 17-day old *An. stephensi *samples. We showed that the miR-14 level increased slightly during embryonic development and remained relatively high through larvae, pupae and adult stages and we did not observe significant changes in adults regardless of age, sex, and blood feeding status. These results do not necessarily imply that mosquito miR-14 is important to longevity, a function of miR-14 demonstrated in *D. melanogaster*. Nonetheless, it appears that miR-14 is important across different mosquito life stages from embryos to aged adults. Further research on the targets and function of miR-14 in mosquitoes will help determine whether it is important to mosquito longevity. The expression profile of miR-x1 through miR-x4 confirmed our cloning results for these new miRNAs, and provided useful information for future research into their functions. The expression of miR-x2 in *An. stephensi *was adult specific as well as female specific. miR-x2 was predominantly expressed in the ovary and its level was reduced 72 hrs after blood feeding. These results indicate that miR-x2 is likely involved in *An. stephensi *female reproduction. The same pattern of expression was shown for miR-x2 in *Ae. aegypti*, a mosquito that is highly divergent from *An. stephensi*. Thus the function of miR-x2 may be conserved among divergent mosquitoes.

We have identified 10 miRNAs previously unknown to mosquitoes, four of which did not match any known miRNAs in any organism. These four miRNAs are conserved between *An. stephensi*, *An. gambiae*, and *Ae. aegypti*, suggesting important functions possibly common to all mosquitoes as the *Anopheles *and *Aedes *genera are two of the most divergent among all mosquitoes, separated approximately 145–200 million years ago [39]. Considering the modest number of small RNA clones we sequenced during this study and the specific developmental stage of our total RNA source, we suspect that a number of novel miRNAs still await to be discovered in mosquitoes. This study demonstrates the importance of direct cloning and expression profile analysis to the identification and characterization of conserved as well as mosquito-specific microRNAs, some of which will likely regulate genes that significantly affect mosquito biology and perhaps mosquito-pathogen interactions.

Three research articles became available when this manuscript was being reviewed. Two of these papers describe additional miRNAs from *Drosophila *[40,41] and a third paper reports cloning of 10 distinct miRNAs and primer extension analysis of an additional 8 miRNAs from *An. gambiae *midgut samples infected with *Plasmodium bergei *[42]. Comparisons between the 27 miRNAs reported in this manuscript and those published in the above three papers showed that *An. stephensi *miR-x1 and miR-x2 are nearly identical to *An. gambiae *miR-996 and miR-989, respectively [42] and homologs of these two miRNAs are found in the expanded list of *Drosophila *miRNAs [40,41]. Thus miR-x1 and miR-x2 are not unique to mosquitoes. Ast-miR-76 showed a perfect match to the newly identified miR-981 in *Drosophila *[40,41], thus ast-miR-76 should be renamed as ast-miR-981. An additional five of the cloned miRNAs reported in Winter *et al*. [42] match five of the 27 miRNAs reported in this manuscript. They are let-7, miR-281, miR-34, miR-12, and miR-306. *An. stephensi *miR-x3 and miR-x4 showed no similarity to any miRNAs in the miRbase which included updates from the above three papers. Thus miR-x3 and miR-x4 remain novel and potentially specific to mosquitoes. The final names of these miRNAs as assigned by miRBase are shown in Table 2. We also compared miRNA expression profiles obtained in our study using northern analysis with the profiles of overlapping miRNAs reported in Winter *et al*. [42], which were obtained using primer extension. Winter and colleagues state that miR-989 (miR-x2) is expressed only in midguts of *An. gambiae *(page 6958). However, we have shown that miR-x2 (miR-989) is predominantly expressed in ovaries in both *An. stephensi *and *Ae. aegypti *and miR-x2 was not detected in midguts under our condition. We note that the Figure 2 of Winter *et al*. [42] does show a much stronger expression in "leftovers", which include ovaries, than in midguts.

## Conclusion

This study provides experimental evidence for 23 conserved and four new microRNAs in *An. stephensi *mosquitoes. Comparisons between miRNA gene clusters in *An. gambiae*, *Ae. aegypti*, and *D. melanogaster *suggest the loss or significant change of two miRNA genes in *Ae. aegypti*. Expression profile analysis of eight miRNAs, including the four new miRNAs, revealed distinct patterns from early embryo to adult stages in *An. stephensi*. Further analysis showed that miR-x2 is likely involved in female reproduction and its function may be conserved among divergent mosquitoes. Consistent expression of miR-14 suggests that miR-14 is likely important across all mosquito life stages from embryos to aged adults. Understanding the functions of mosquito miRNAs will undoubtedly contribute to a better understanding of mosquito biology including longevity, reproduction, and mosquito-pathogen interactions, which are important to disease transmission.

## Methods

### Mosquitoes

*An. stephensi *(Indian wild-type strain) and *Ae. aegypti *(Liverpool strain) mosquitoes were maintained in humidified incubators at 27°C on a 12 hour light:dark cycle.

### Small RNA cloning and sequencing

For small RNA cloning, approximately 1000 female *An. stephensi *adults were collected at 17 days post-emergence. Mosquitoes were fed blood and allowed to oviposit prior to collection. Aged females are the most relevant mosquito life stage for malaria transmission as it takes approximately two weeks for *Plasmodium *parasites to mature within the mosquito to an infective stage [24]. RNA was isolated using a mirVana small RNA Isolation Kit (Ambion, Austin, TX). Subsequent steps were performed following published protocols [6] from the David Bartel laboratory (Whitehead Institute, MIT, Cambridge, MA). RNA oligo markers (18-mer, 24-mer; sequence as detailed from [6]) were purchased from Dharmacon (Lafayette, CO) and 2' ends were deprotected prior to use as indicated by Dharmacon. These oligos were end-labeled with ^32^P (^32^P-γ ATP, Perkin-Elmer, Waltham, MA) by T4 polynucleotide kinase (Promega, Madison, WI) and added to the sample to isolate RNAs between 18 and 24 nt in length. Next, 5' and 3' linkers were sequentially ligated to the isolated small RNA as well as the RNA markers. cDNA were generated by RT-PCR using primers derived from the two linker sequences. cDNA were ligated into a 2.1 TOPO TA vector (Invitrogen, Carlsbad, CA). Ligated plasmids were transformed in One-Shot Mach 1-T1^R ^Competent cells (Invitrogen). We did not concatemerize cDNA prior to cloning. Sequencing was performed by Virginia Bioinformatics Institute core facilities and the University of Washington High Throughput Genomics Unit.

### Small RNA Sequence Analyses

Sequences obtained from cloning were analyzed with ClustalW [43] to identify inserts and orientation. After removing low quality sequences determined by inspection of chromatographs, insert sequences were analyzed using BLAST [44] against different databases as described below to identify matches to known miRNAs, mRNAs, tRNAs, rRNA, and other small RNAs (Table 1). Sequences that were identical to previously predicted *An. gambiae *miRNAs were identified by comparing with the miRBase miRNA registry [25] and *An. gambiae *miRNA predictions listed in [23] using BLAST [44]. Comparisons to miRBase also revealed sequences that match miRNAs from other organisms but were not reported in mosquitoes. The remaining *An. stephensi *small RNA sequences that matched the *An. gambiae *genome assembly were further analyzed to uncover new miRNAs from mosquitoes (Table 2). *An. gambiae *genome assembly was used to retrieve miRNA precursors for secondary structural analysis because there was little sequence information available for the *An. stephensi *genome and the two species are in the same subgenus *Celia*. The matching *An. gambiae *sequences plus 100 nt flanking sequences were obtained through Ensembl [45] and lowest energy conformations were generated by Vienna RNAfold [46]. The above mentioned *An. gambiae *precursor sequences were used to search for conserved sequences in *Ae. aegypti*, and in some cases, in *D. melanogaster*. Conserved sequence pairs were then examined via miRscan [28]. One *An. stephensi *miRNA (ast-miR-304) was unscorable by miRscan (Table 2) and the precursor of this miRNA was examined using the biogenesis criteria described in the literature: 1) the miRNA appears in the stem of a hairpin structure; 2) there is considerable similarity between the miRNA and miRNA*; and 3) few bulges if any are present within the miRNA:miRNA* pairing in the stem [31,32].

### Northern blot

Northern hybridizations shown in Figure 1 were conducted using miRCURY-LNA probes from Exiqon (Vedbaek, Denmark) for eight different miRNAs. Total RNA was isolated from *An. stephensi *of the same age or developmental stage and used for each blot. Ten micrograms of total RNA was loaded for each sample in a 15% denaturing polyacrylamide gel. After initial size confirmation using let-7 RNA, 19 nt and 23 nt single-stranded DNA oligos were used as size markers in subsequent experiments. Gels were stained with ethidium bromide for verification of equal loading and RNA quality before transferring to a BrightStar-Plus membrane (Ambion). Subsequent steps were based upon Wienholds *et al*. [47]. Following UV crosslinking, membranes were prehybridized in a rotating oven for 30 minutes at 42°C using ULTRAhyb-Oligo Hybridization Buffer (Ambion), followed by overnight hybridization in the same buffer at 42°C with a final concentration of 0.1 nM digoxigenin (DIG)-labeled antisense miRCURY LNA probe (Exiqon). All probes used in northern blots were designed to hybridize with *An. stephensi *miRNAs. The membranes were washed twice for 35 minutes in an Ambion-recommended wash buffer (2 × SSC, 0.5% SDS) at 42°C, and then once in low stringency buffer for 5 minutes at room temperature. The membranes were incubated for 30 minutes in blocking buffer followed by a 1 hr incubation with Anti-DIG-alkaline phosphatase fAb (Roche, Basel, Switzerland) in blocking buffer. The membranes were washed three times for 15 minutes in low stringency wash buffer followed by twice with alkaline phosphatase buffer. The membranes were then immersed in CDP-Star solution (Roche) for 5 minutes, and placed inside saran wrap for exposure to X-ray film for 30 minutes.

For the northern blots shown in Figure 5, a similar procedure was followed. The samples were 3, 5, 10, 17, and 24 day old *An. stephensi *adult females as well as 3, 5, 10, and 17 day old *An. stephensi *adult males that were maintained on sugar water. The same cohort of adult females, which were fed on blood on day 5 after emergence and allowed to oviposit two days later, were collected at day 10, 17, and 24. RNA isolation and northerns were carried out as detailed above. For tissue sample northern blots (Figure 6), five day old adult *An. stephensi *females were split into two groups, one maintained on sugar water, the other provided a blood meal then maintained on sugar water. Approximately twenty-four hrs post bloodfeeding, heads, ovaries, midguts and remainders (everything left behind) were collected from 60 bloodfed and 60 non-bloodfed six day old mosquitoes. This procedure was repeated again at 72 hrs after blood feeding with eight day old mosquitoes. The mosquitoes were not permitted to oviposit. All tissues were stored in RNA *later *(Ambion) during collection, then vortexed and stored in -80°C until RNA isolation as above using a *mir*Vana miRNA Isolation kit (Ambion). Northern hybridization was performed as describe above except that 5 ug of total RNA were used. The same procedure was followed for *Ae. aegypti *mosquito tissue analysis. In separate experiments, 10 ug of total RNA isolated from whole body were used to compare the expression of miR-x2 in males and non-bloodfed females of both species. We have performed replicates for all expression profile analyses and obtained consistent expression patterns between replicates.

### Ribonuclease Protection Assay (RPA)

RPA was used to examine ast-miR-76. Double-stranded oligos with a T7 promoter 5' of an antisense sequence of the miRNA were produced by annealing two single-stranded oligos. The annealed oligos were used to synthesize an RNA probe that was 9 nt longer than the miRNA, as seen below:

ast-miR-76

5'TTCGTTGTCGACGAAACCTGTTTTCTCCCTATAGTGAGTCGTATTA 3'

3'AAGCAACAGCTGCTTTGGACAAAAGAGGGATATCACTCAGCATAAT 5'

Our design also considered the possibility for future multiplexing by adding extra 4 adenosines immediately following +1 which were indigestible according to the instruction manual of the "mirVana miRNA Probe Construction Kit" (Ambion). This permitted the undigested probe to run at ~29 nt, and digested/protected probe to run at ~24 nt. We utilized a "MEGAscript RNAi kit" (Ambion) to synthesize radiolabeled (^32^P-UTP, Perkin-Elmer) antisense transcripts. Templates were synthesized for 4 hours, and purified from acrylamide gels by elution and isopropanol precipitation (1× volume) with 1/10 volume 3 M NaOAc and glycogen. 50,000 cpm of probe was added to 5 ug total RNA and denatured at 96°C for 3 minutes followed by 42°C hybridization overnight. Next, the hybridized samples were digested with RNase A/T1 for 45 minutes at 37°C to remove single-stranded RNA, and to trim unhybridized regions of the probes. Afterwards, the samples were ethanol precipitated and resuspended in 5 ul 2 × loading buffer. Resuspended samples were denatured at 96°C for 5 minutes followed by a quick chill on ice before loading. RNA were separated using a 15% denaturing polyacrylamide gel at 150 V. Locations of bands were examined by X-ray film exposure, using an intensifying screen. Size markers were as described for northern blot analysis.

## Abbreviations

digoxigenin: DIG; Locked nucleic acid: LNA; microRNA: miRNA; nucleotide: nt; untranslated region: UTR.

## Authors' contributions

EAM assisted in experimental design. EAM conducted the experimental work, analyzed data, and wrote a rough draft of the manuscript. ZT prepared the experimental design, helped with data analysis, and revised the manuscript; ZT is the principal investigator who oversaw this project.

## Supplementary Material

Additional file 1 Click here for file

## References

[B1] Miska EA (2005). How microRNAs control cell division, differentiation and death. Curr Opin Genet Dev.

[B2] Du T, Zamore PD (2007). Beginning to understand microRNA function. Cell Research.

[B3] Bartel DP (2004). MicroRNAs: genomics, biogenesis, mechanism, and function. Cell.

[B4] Pasquinelli AE, Hunter S, Bracht J (2005). MicroRNAs: a developing story. Curr Opin Genet Dev.

[B5] Zhou X, Ruan J, Wang G, Zhang W (2007). Characterization and identification of MicroRNA core promoters in four model species. PLoS Comput Biol.

[B6] Lau NC, Lim LP, Weinstein EG, Bartel DP (2001). An Abundant Class of Tiny RNAs with Probable Regulatory Roles in Caenorhabditis elegans. Science.

[B7] Denli A, Tops BBJ, Plasterk RHA, Ketting RF, Hannon GJ (2004). Processing of primary microRNAs by the Microprocessor complex. Nature.

[B8] Bushati N, Cohen SM (2007). microRNA Functions. Annual Review of Cell and Developmental Biology.

[B9] Brennecke J, Stark A, Russell RB, Cohen SM (2005). Principles of microRNA-target recognition. PLoS Biol.

[B10] Grimson A, Farh KK-H, Johnston WK, Garrett-Engele P, Lim LP, Bartel DP (2007). MicroRNA Targeting Specificity in Mammals: Determinants beyond Seed Pairing. Mol Cell.

[B11] Lai EC (2005). miRNAs: Whys and Wherefores of miRNA-Mediated Regulation. Curr Biol.

[B12] Du T, Zamore PD (2005). microPrimer: the biogenesis and function of microRNA. Development.

[B13] Kim VN (2005). Small RNAs: Classification, Biogenesis, and Function. Mol Cells.

[B14] Ambros V, Chen X (2007). The regulation of genes and genomes by small RNAs. Development.

[B15] O'Connell RM, Taganov KD, Boldin MP, Cheng G, Baltimore D (2007). MicroRNA-155 is induced during the macrophage inflammatory response. PNAS.

[B16] le Sage C, Nagel R, Egan DA, Schrier M, Mesman E, Mangiola A, Anile C, Maira G, Mercatelli N, Ciafrè SA (2007). Regulation of the p27Kip1 tumor suppressor by miR-221 and miR-222 promotes cancer cell proliferation. EMBO J.

[B17] Cullen BR (2006). Viruses and microRNAs. Nature Genetics.

[B18] Nakahara K, Kim K, Sciulli C, Dowd SR, Minden JS, Carthew RW (2005). Targets of microRNA regulation in the Drosophila oocyte proteome. PNAS.

[B19] Lewis BP, Burge CB, Bartel DP (2005). Conserved Seed Pairing, Often Flanked by Adenosines, Indicates that Thousands of Human Genes are MicroRNA Targets. Cell.

[B20] Lai E, Tomancak P, Williams R, Rubin G (2003). Computational identification of Drosophila microRNA genes. Genome Biology.

[B21] Aravin AA, Lagos-Quintana M, Yalcin A, Zavolan M, Marks D, Snyder B, Gaasterland T, Meyer J, Tuschl T (2003). The Small RNA Profile during Drosophila melanogaster Development. Dev Cell.

[B22] Wang X, Zhang J, Li F, Gu J, He T, Zhang X, Li Y (2005). MicroRNA identification based on sequence and structure alignment. Bioinformatics.

[B23] Chatterjee R, Chaudhuri K (2006). An approach for the identification of microRNA with an application to Anopheles gambiae. Acta Biochim Pol.

[B24] U.S. Centers for Disease Control. http://www.cdc.gov/malaria/biology.

[B25] The miRBase Registry. http://microrna.sanger.ac.uk.

[B26] Xu P, Vernooy SY, Guo M, Hay BA (2003). The Drosophila microRNA Mir-14 suppresses cell death and is required for normal fat metabolism. Curr Biol.

[B27] Valoczi A, Hornyik C, Varga N, Burgyan J, Kauppinen S, Havelda Z (2004). Sensitive and specific detection of microRNAs by northern blot analysis using LNA-modified oligonucleotide probes. Nucl Acids Res.

[B28] miRscan Web Server. http://genes.mit.edu/mirscan.

[B29] Lim LP, Lau NC, Weinstein EG, Abdelhakim A, Yekta S, Rhoades MW, Burge CB, Bartel DP (2003). The microRNAs of Caenorhabditis elegans. Genes Dev.

[B30] Lim LP, Glasner ME, Yekta S, Burge CB, Bartel DP (2003). Vertebrate MicroRNA Genes. Science.

[B31] Ambros V, Bartel B, Bartel DP, Burge CB, Carrington JC, Chen X, Dreyfuss G, Eddy SR, Griffiths-Jones SAM, Marshall M, Matzke M, Ruvkun G, Tuschl T (2003). A uniform system for microRNA annotation. RNA.

[B32] Gu Z, Eleswarapu S, Jiang H (2007). Identification and characterization of microRNAs from the bovine adipose tissue and mammary gland. FEBS Lett.

[B33] Griffiths-Jones S (2004). The microRNA Registry. Nucl Acids Res.

[B34] Griffiths-Jones S, Grocock RJ, van Dongen S, Bateman A, Enright AJ (2006). miRBase: microRNA sequences, targets and gene nomenclature. Nucl Acids Res.

[B35] Smalheiser NA (2003). EST analyses predict the existence of a population of chimeric microRNA precursor-mRNA transcripts expressed in normal human and mouse tissues. Genome Biol.

[B36] Kim YK, Kim VN (2007). Processing of intronic microRNAs. EMBO J.

[B37] Sempere LF, Sokol NS, Dubrovsky EB, Berger EM, Ambros V (2003). Temporal regulation of microRNA expression in Drosophila melanogaster mediated by hormonal signals and broad-Complex gene activity. Dev Biol.

[B38] Berezikov E, Cuppen E, Plasterk RHA (2006). Approaches to microRNA discovery. Nat Genet.

[B39] Krzywinski J, Grushko OG, Besansky NJ (2006). Analysis of the complete mitochondrial DNA from Anopheles funestus: An improved dipteran mitochondrial genome annotation and a temporal dimension of mosquito evolution. Mol Phylogenet Evol.

[B40] Ruby JG, Stark A, Johnston WK, Kellis M, Bartel DP, Lai EC (2007). Evolution, biogenesis, expression, and target predictions of a substantially expanded set of Drosophila microRNAs. Genome Res.

[B41] Stark A, Kheradpour P, Parts L, Brennecke J, Hodges E, Hannon GJ, Kellis M (2007). Systematic discovery and characterization of fly microRNAs using 12 Drosophila genomes. Genome Res.

[B42] Winter F, Edaye S, Huttenhofer A, Brunel C (2007). Anopheles gambiae miRNAs as actors of defence reaction against Plasmodium invasion. Nucl Acids Res.

[B43] Thompson JD, Higgins DG, Gibson TJ (1994). improving the sensitivity of progressive multiple sequence alignment through sequence weighting, position- specific gap penalties and weight matrix choice. Nucl Acids Res.

[B44] Altschul SF, Madden TL, Schaffer AA, Zhang J, Zhang Z, Miller W, Lipman DJ (1997). Gapped BLAST and PSI-BLAST: a new generation of protein database search programs. Nucl Acids Res.

[B45] Ensembl. http://www.ensembl.org.

[B46] Vienna RNA Secondary Structure Prediction. http://rna.tbi.univie.ac.at/cgi-bin/RNAfold.cgi.

[B47] Wienholds E, Kloosterman WP, Miska E, Alvarez-Saavedra E, Berezikov E, de Bruijn E, Horvitz HR, Kauppinen S, Plasterk RHA (2005). MicroRNA Expression in Zebrafish Embryonic Development. Science.

